# Supplementary material for: Fully Printed Zinc Oxide Electrolyte-Gated Transistors on Paper
Source: Nanomaterials (Basel). 2019 Jan 30;9(2):169. doi: 10.3390/nano9020169 (PMC6410167; doi:10.3390/nano9020169)
Supplement: Supplementary file 1 [file nanomaterials-09-00169-s001.pdf]

Supporting Information

Fully Printed Zinc Oxide Electrolyte-Gated Transistors on Paper

José Tiago Carvalho [1], Viorel Dubceac [1], Paul Grey [1], Inês Cunha [1], Elvira Fortunato [1], Rodrigo Martins [1], Andre Clausner [2], Ehrenfried Zschech [2], and Luís Pereira [1,*]

[1] CENIMAT/I3N, Departamento de Ciência dos Materiais, Faculdade de Ciências e Tecnologia, FCT, Universidade Nova de Lisboa and CEMOP-UNINOVA, Campus da Caparica, 2829-516 Caparica, Portugal; jt.carvalho@campus.fct.unl.pt (J.T.C.); v.dubceac@campus.fct.unl.pt (V.D.); paul16_grey@yahoo.de (P.G.); i.cunha@campus.fct.unl.pt (I.C.); emf@fct.unl.pt (E.F.); rfpm@fct.unl.pt (R.M.)

[2] Fraunhofer Institute for Ceramic Technologies and Systems (IKTS), 01109 Dresden, Germany; andre.clausner@ikts.fraunhofer.de (A.C.); ehrenfried.zschech@ikts.fraunhofer.de (E.Z.)

* Correspondence: lmnp@fct.unl.pt

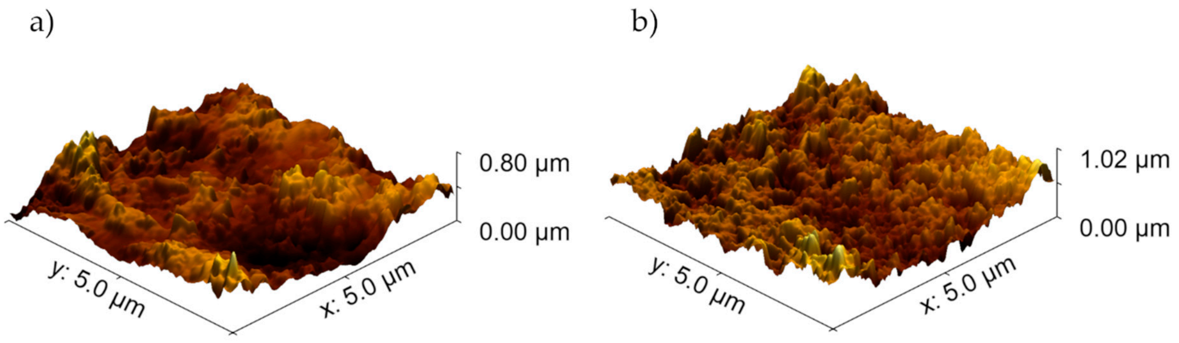

Figure S1. AFM topographic images (without being annealed) of a) screen-printed ZnO10 ink and b) screen-printed ZnO40.

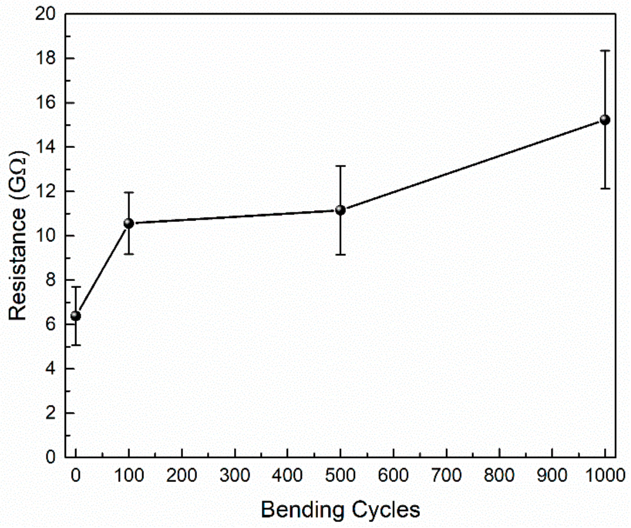

Figure S2. Dependence of the resistance on the number of bending cycles, for screen-printed ZnO40 films on paper.

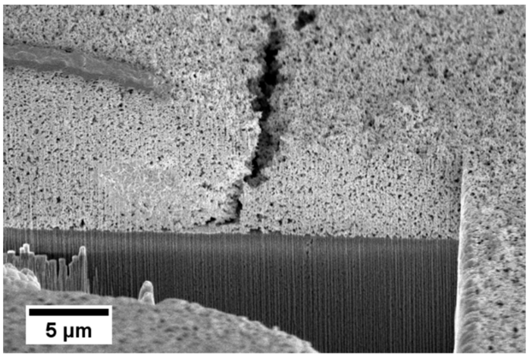

Figure S3. Cross section image of the ZnO40 screen-printed film on paper, after being subjected to 1000 bending cycles. The cross section was prepared by FIB.

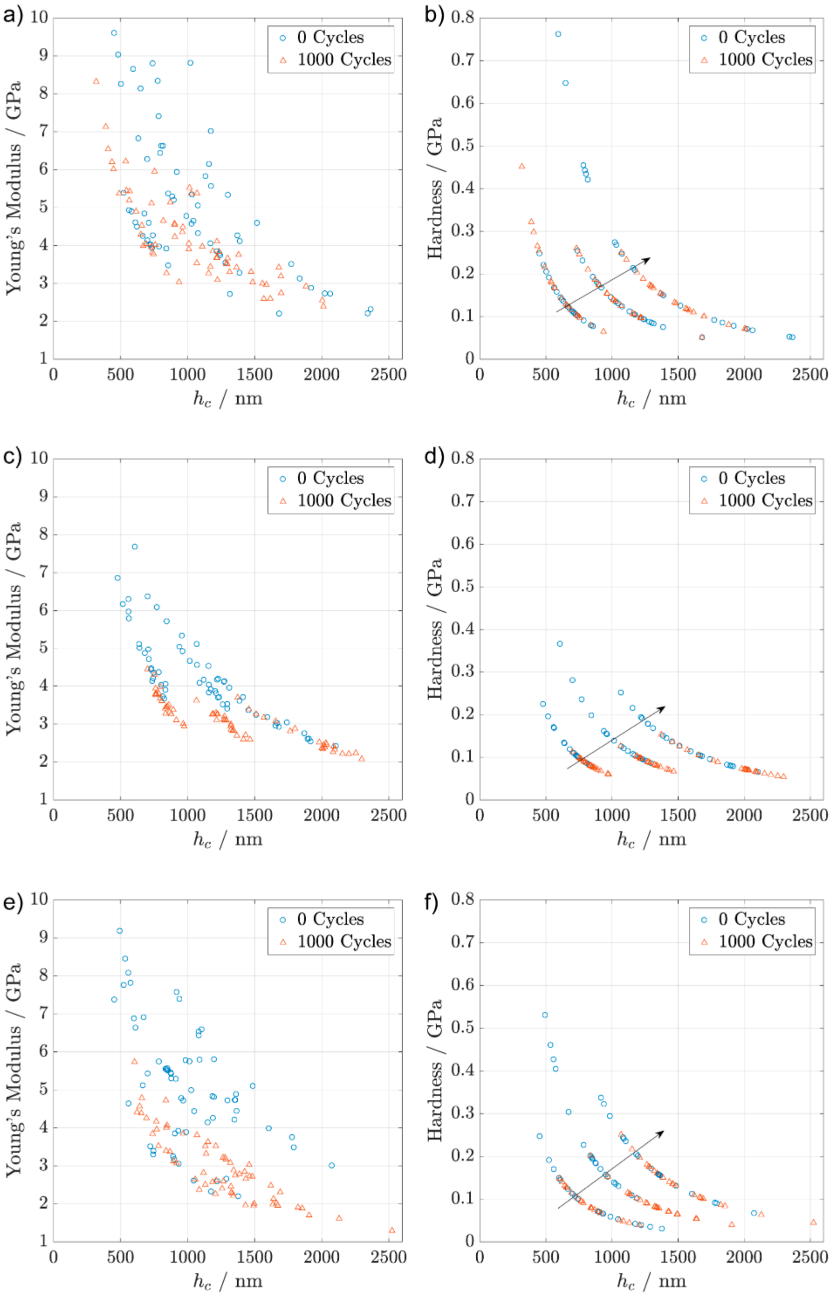

Figure S4. Young´s Modulus and Hardness results obtained for 3 different regions, for screen-printed ZnO40 films on paper after being subjected to the bending cycles.

Characterization of the Composite Solid Polymer Electrolyte

Electrochemical impedance spectroscopy (EIS) of the composite solid polymer electrolyte (CSPE) was performed between two stainless steel electrodes with a cell area of A = 1.04 cm^2, as depicted in Figure S5a. EIS was carried out with a peak-to-peak *ac* Voltage of 25 mV (in respect to an open circuit potential (OCP) of 1 V) in a frequency range from 10^{-1} to 10^6 Hz. The quantification of the dielectric parameters was performed by fitting of an equivalent circuit model (ECM), as shown in Figure S5b. The ECM simulates an ideally polarizable electrochemical cell, to describe the obtained data set in the applied frequency spectrum. From the EIS fitting, the C_{DL}, was determined as 2.57 × 10^{-6} F, with an Y_0 of 2.15 × 10^{-5} Ss^{α}, R_{ext} equal to 117.2 Ω, α equal to 7.38 × 10^{-1}. The C_{DL} per area corresponds to 2.47 × 10^{-6} Fcm^{-2}, considering a cells area of 1.04 cm^2.

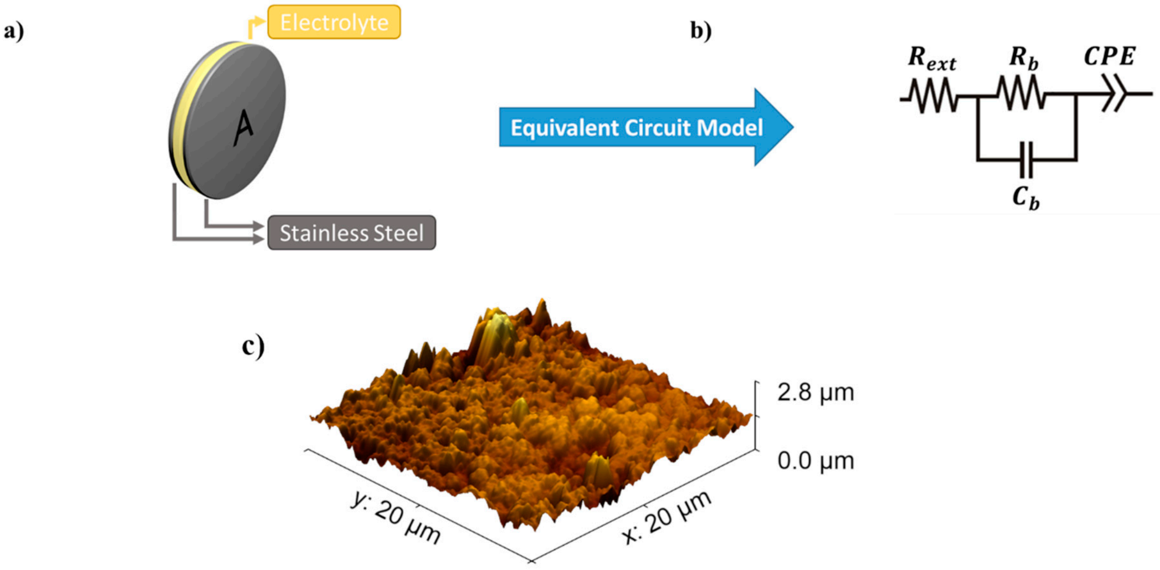

Figure S5. a) Schematic representation of the electrochemical cell setup with an area (A) of 1.04 cm^2 and b) the corresponding ECM suggested by Dasgupta *et al.* adapted from [1] where R_{ext} is contact resistance, R_b and C_b corresponds to the electrolyte resistance and capacitance, respectively, and CPE the constant phase element, c) the AFM topographic image of the screen-printed composite solid polymer electrolyte (CSPE).

The presence of stainless-steel electrodes is essential for the conducted analysis, as they minimize undesired redox reactions between the accumulated ions and the electrode material, which could result in faradaic currents. The use of practically inert electrode materials gives rise to capacitive currents predominantly, as a result of charge induction caused by the ion migration process and charge transfer into the neighboring electrodes. Consequently, the ECM is composed of three basic building blocks. R_{ext} is the external contact resistance and associated to the stainless-steel electrodes and cable contacts. Moreover, it also models part of the non-ideal capacitive behavior of the C_{DL} (such as ionic diffusion across the interface). The parallel RC circuit is intrinsically connected to the electrolyte bulk behavior, where R_b and C_b are its resistance and capacitance, respectively. Here C_b is mainly associated to a dipolar relaxation mechanism of solvent molecules in the electrolyte at very high frequencies[1] of more than 10^4 Hz. It produces negligible values compared to the double layer capacitance (C_{DL}). Also, in the context of the characterized devices, the frequencies for which this mechanism takes place are too high to be considered. The last component of the ECM is connected in series with the other two and consists of a constant phase element (CPE). Since the ions migrate freely throughout the electrolyte bulk they follow the applied signal. At sufficiently low frequencies they start to accumulate at the electrode/electrolyte interfaces (ionic relaxation), giving rise to high electric fields at the interfaces. This process originates electric double layers (EDL), forming a parallel plate capacitor at each interface with a thickness of merely a few Angstroms. The EDL consequently transfers the ionic charge into the neighboring material, being the fundamental

concept behind electrolyte gating in transistors. However, owing to possible interface inhomogeneities, originating from high surface roughness of the electrolyte (Figure S5c, Supporting Information), the behavior can no longer be modeled by a pure capacitor. Hence, the CPE mimics a non-ideal leaky capacitive behavior of the EDL at the interfaces with non-uniform current distribution[2].

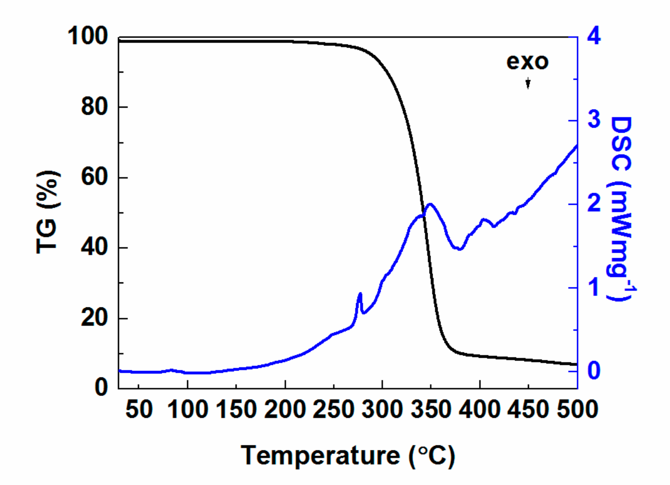

Figure S6. DSC-TG curves performed in air up to 500 °C for the Ethyl Cellulose (EC) powder.

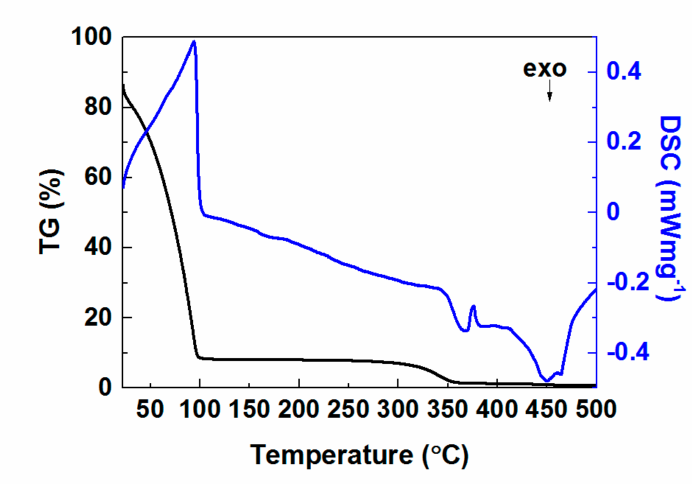

Figure S7. DSC-TG curves performed in air up to 500 °C for the binder solution (ethyl cellulose 5 wt% dissolved in a solvent mixture of toluene:ethanol (80:20 %v/v).

Considerations about saturation mobility determination.

The saturation mobility (μ_{Sat}) of the devices was determined through equation S1 [3]

$$\mu_{Sat} = \frac{\left(\frac{d\sqrt{I_D}}{dV_G}\right)^2}{\frac{1}{2}C_i\frac{W}{L}} \qquad \text{(S1)}$$

where $\left(\frac{d\sqrt{I_D}}{dV_G}\right)^2$ is transconductance (gm), W and L corresponds to the width and length of the channel; C_i is the capacitance of the dielectric layer, which in this case corresponds to the C_{DL} (2.47×10^{-6} Fcm^{-2}).

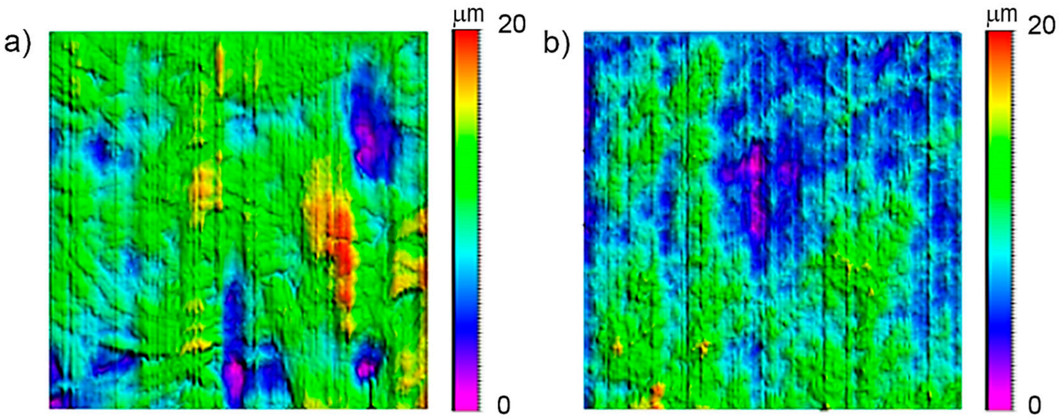

Figure S8. The 3D profilometry scan of the surface of the a) printing paper and b) MFC kraft substrates.

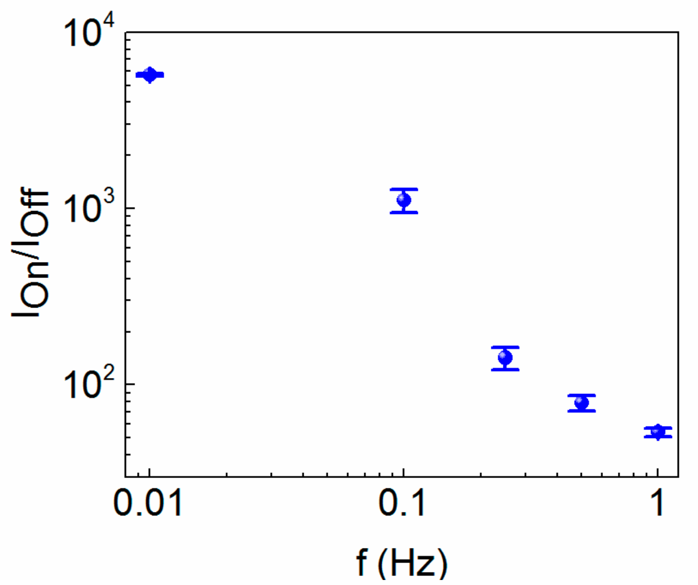

Figure S9. Dynamic electrical characterization of the $ZnO40_{150\,°C\,MK}$ EGT: variation of the I_{On}/I_{Off} using a square-shaped V_G (V_{DS}= 1.3 V, 5 cycles for each frequency 0.01, 0.1, 0.25, 0.5 and 1 Hz).

References

1. Dasgupta, S.; Stoesser, G.; Schweikert, N.; Hahn, R.; Dehm, S.; Kruk, R.; Hahn, H. Printed and Electrochemically Gated, High-Mobility, Inorganic Oxide Nanoparticle FETs and Their Suitability for High-Frequency Applications. *Adv. Funct. Mater.* **2012**, *22*, 4909–4919, doi:10.1002/adfm.201200951.

2. Barsoukov, E.; Macdonald, J.R. Physical Models for Equivalent Circuit Elements. In *Impedance Spectroscopy: Theory, Experiment, and Applications*; John Wiley & Sons, Inc: New Jersey, 2005; p. 13 ISBN 9780471716242.

3. Santos, L.; Nunes, D.; Calmeiro, T.; Branquinho, R.; Salgueiro, D.; Barquinha, P.; Pereira, L.; Martins, R.; Fortunato, E. Solvothermal Synthesis of Gallium–Indium-Zinc-Oxide Nanoparticles for Electrolyte-Gated Transistors. *ACS Appl. Mater. Interfaces* **2015**, *7*, 638–646, doi:10.1021/am506814t.
